# Effect of kesse, koseret, and tosign extract treatments on the oxidative stability of oil during the repeated frying of potato chips

**DOI:** 10.1016/j.heliyon.2024.e36868

**Published:** 2024-08-26

**Authors:** Daniel Assefa, Engida Dessalegn, Kebede Abegaz

**Affiliations:** a Department of Chemistry, College of Natural and Computational Sciences, Dilla University, Dilla, Ethiopia; b Department of Chemistry, Hawassa College of Teacher Education, Hawassa, Ethiopia; c School of Nutrition, Food Science, and Technology, Hawassa University, Hawassa, Ethiopia

**Keywords:** Frying cycles, Natural antioxidants, Herb extracts, Oil stability, Sensory properties

## Abstract

Fried food safety and quality are crucial concerns for consumers and the food industry due to the deterioration of oil quality and the loss of sensory properties during repeated frying. The current study investigated the use of leaf extracts from endemic dietary herbs: kesse (*Lippia adoensis* var. adoensis), koseret (*Lippia adoensis* var. koseret), and tosign (*Thymus schimperi* Ronninger) to enhance oil quality during the repeated frying of potato chips. The analysis of various parameters, including free fatty acids (FFA), iodine value (IV), peroxide value (PV), thiobarbituric acid reactive substances (TBARS), viscosity, and color (L*, a*, and b*), was conducted after every 5th frying cycle to assess oil quality. The results revealed significant (p < 0.05) decreases in deterioration markers for oils treated with herb extracts compared to the control oils. Specifically, after 20 frying cycles, oils treated with the dietary herb extracts exhibited lower percentage of FFA (0.63–1.05), IV (51.7–46.7), PV (6.69–7.68), and TBARS (50.27–56.08) compared to the control. The herb-treated oils also maintained lower FFA, PV, and TBARS values throughout the frying cycles and reduced viscosity, while IV gradually decreased. Furthermore, the L* value decreased gradually, and a* and b* values increased as the number of frying cycles increased. The herb extracts provided better protection against degradation compounds compared to BHT-treated and control oils, which was attributed to their lower FFA and PV. Sensory analysis indicated that potato chips fried in kesse extract-treated oil were the most preferred, followed by those treated with tosign extract. These findings highlight the potential application of herb extracts to increasing oil stability during repeated frying cycles, which add value at the interface between culinary excellence and health perspectives. Natural antioxidants from endemic herbs can maintain oil quality, reduce harmful compounds, and enhance the sensory properties of fried foods, making them a promising alternative to synthetic antioxidants.

## Highlights

• The effect of kesse, koseret, and tosign extracts on oil quality during multiple frying cycles was investigated.
• Dietary herb extracts significantly reduce markers of oil deterioration compared to control oils.
• Oil color varied in L*, a*, and b* values due to repeated frying cycles.
• Kesse extract-treated oil produced the most desirable potato chips, followed by oil treated with tosign extract.
• Sensory analysis indicated that potato chips fried in kesse extract-treated oil were the most preferred.

## 1 Introduction

Frying is a popular cooking method for many foods, such as potato chips, dough, and chicken wings. However, the safety of frying oil has become a significant concern globally due to its widespread use in the food industry and the increased production of deterioration products during frying [1]. The chemical processes involved in frying such as oxidation, hydrolysis, isomerization, and polymerization result in a decline in the nutritional quality of both the frying oils and the foods being prepared [2,3]. Nayak et al. [4] suggested that during frying, the breakdown and oxidation of oils lead to the formation of free fatty acids, carbonyl compounds and low molecular weight fatty acids. These chemical reactions produce undesirable changes that ultimately result in oil deterioration and a reduced shelf life for frying [5].

Research has also shown that deep frying can negatively impact human health due to the absorption of both primary and secondary derivatives of the oil. This absorption can lead to the formation of free radical and the production of the toxic compounds, which may increases the rates of aging and the risk of degenerative disease associated with oxidative stress [4,6]. Meanwhile, frying under optimal conditions may lead to the formation of some desirable compounds responsible for appealing flavor, taste, crispy texture, and characteristic golden color of fried foods [7]. As a result, vegetable oils are widely used as cooking oils, particularly for frying, to improve organoleptic properties and enhance the overall palatability of food products [8,9].

Street vendors and fast-food restaurants often utilize the same oil for an extended period and multiple frying cycles to reduce costs over time. However, these practices can lead to the formation of free radicals and oxidation of the oil, resulting in deterioration of the oil quality that may produce undesired flavors and harmful compound [6,10]. The continued use of oil in frying poses a significant risk to both the quality of the oil and the fried food itself [10]. Such practices can result in the formation of potentially harmful compounds, including heterocyclic amines, acrylamides, furfurals, and advanced glycation end products [11,12]. These toxic compounds can

Accumulate to high concentrations through repeated frying and the reuse of cooking oil [13].

As reported by Dourado et al. [14] and Lacoste et al. [15], the addition of antioxidants is the most direct and frequently used method to extend the shelf life of frying oils, retard the formation of undesirable components, and reduce the formation of trans fatty acids [16,17]. Both synthetic and natural antioxidants have been employed to stabilize frying oils against thermal lipid oxidation. However, consumer preferences are increasingly leaning toward natural options, making synthetic antioxidants less desirable [18]. This shift has led to growing interest in naturally derived antioxidants for frying applications. Many constituents of plant extracts, like flavonoids and phenolic acids, exhibit bioactive properties that confer antioxidant benefits [19]. The presence of these compounds can inhibit or slow down the oxidation of oils and fats, thereby improving the oxidative stability of frying oils.

The use of natural antioxidants is an effective approach to improve the stability of frying oil without compromising its quality parameters [20]. Furthermore, there are several techniques for incorporate antioxidant from herbs into oil, including the use of crude extracts, essential oils, or maceration of the herbs directly in the oil. The oil-soluble active compounds in herbs possess antioxidant properties that contribute to preserving the stability of oil during usage [21].

*Lippia adoensis* var. koseret, known locally in Ethiopia as koseret, is a valued herb recognized for its aromatic properties and traditional medicinal uses in treating infectious diseases and enhancing spiced butter [22,23]. Extracts from this plant contain volatile oils that are commonly used to flavor Ethiopian dishes like "Gurage Kitfo” which is made from minced raw beef with herb-infused clarified butter [24]. Shiferaw et al. [23] found that koseret leaf oil exhibits better antioxidant and antibacterial properties compared to seed oil, due to its geranial and neral constituents, which also impart a pleasant aroma.

*Lippia adoensis* var. adoensis, locally known as kesse, is traditionally used to wash wooden and ceramic kitchen utensils, imparting a lemon-like fragrance. Its refreshing and pungent aroma helps ensure that the butter used in cooking remains fresh and resists rancidity due to its antioxidant effect [25]. The essential oil of kesse contains limonene, along with the ketones piperitenone and perillaldehyde, which are absent in koseret oil [26].

*Thymus schimperi* Ronninger, commonly known as tosign, is a multipurpose wild endemic plant in Ethiopia, recognized for its significant antioxidant activity and traditional medicinal characteristics [27]. The leaves of tosign are very strong natural antioxidants that can effectively stabilize refined soybean oil and butter [28]. In a recent study using asparagine-glucose models, we demonstrated the potential of naturally occurring antioxidants extracted from kesse, koseret, and tosign as promising agents for reducing the formation of acrylamide [29].

This research investigated the effects of leaf extracts from kesse, koseret, and tosign on the physicochemical properties and quality of frying oil, focusing on parameters such as free fatty acid (FFA), peroxide value (PV), iodine value (IV), thiobarbituric acid reactive substances (TBARS), color, and viscosity throughout multiple frying cycles. Additionally, sensory evaluations were also conducted to assess the quality of the fried potato chips.

## 2 Materials and methods

### 2.1 Chemicals and reagents

Malondialdehyde standard, n-Hexane (97.09 % purity), 2-thiobarbituric acid reagent (TBA), BHT, and Wijs solution were purchased from Sigma-Aldrich. Sodium thiosulfate, iodine, acetic acid, chloroform, hydrochloric acid, sodium chloride, ethanol, methanol, acetone, sodium hydroxide, and potassium hydroxide were also obtained from Merck (Darmstadt, Germany).

### 2.2 Herb Collection and preparation

Samples of kesse and tosign were collected from Dinsho, Bale National Park, located 370 km southeast of Addis Ababa, Ethiopia. Koseret samples were collected from Wondo Genet Agriculture Research Center, located 25 km northeast of Hawassa, Ethiopia as part of the study. The herbs utilized in this study were validated by the Herbarium of the Department of Botany at Hawassa University. The plant materials were placed in plastic bags, transported to the laboratory, and thoroughly cleaned by rinsing with distilled water. The cleaned herbs were air-dried in the shade for two weeks, with continuous turning to prevent fungal growth. After drying, the herb leaves were homogenized and reduced to a fine powder using a laboratory mill to ensure effective solvent contact during extraction. Additionally, Durame potato tubers, a variety of potato commonly used for making chips in Ethiopia, along with palm oil for frying, were purchased from local markets and a wholesaler in Hawassa, Ethiopia.

### 2.3 Preparation and extraction of dietary herb

To prepare the extract, 25 g of powdered dried kesse, koseret, and tosign leaves were mixed with 250 mL of 98 % ethanol in separate flasks. The mixtures were continuously shaken at 120 rpm using an orbital shaker (Heidolph UNIMAX 2010, Germany) for 8 h at room temperature. The extracts were then filtered through Whatman No. 1 filter paper. The solvent was removed by evaporation using a rotary evaporator (Buchi, 3000 series, Switzerland) at 50 °C, and the obtained extracts were stored in sealed polyethylene containers at 4 °C until further analysis.

### 2.4 Preparation of potato chips

In accordance with the methodology outline by Guo et al. [30], potato slices were fried in 2.5 L of oil using a thermostat-controlled electric fryer. The oil was supplemented with dietary herb extracts at a concentration of 120 mg/kg and synthetic antioxidants (BHT) at a concentration of 200 mg/kg. Notably, the concentration of BHT adheres to the legal limit of synthetic antioxidants in most countries, according to the Food and Drug Administration. A control sample used the same volume of oil without any added dietary herbs or BHT, and Over a span of two days, batches of potato slices weighing 150 g each were fried repeatedly for two consecutive morning-afternoon cycles, resulting in a total of ten frying cycles per day and 20 frying cycles overall. The frying process utilized consistent parameters (170 °C for 6 min) without replacing the oil. Analysis was conducted on fresh oil samples and samples obtained after the 5th, 10th, 15th, and 20th frying cycles. To facilitate further analysis, 100 mL of oil from each sample was collected and stored in sealed amber-colored glass bottles at 4 °C. Additionally, a batch of the potato chips was designated for sensory evaluation.

### 2.5 Physicochemical measurements of oil

#### 2.5.1 Free fatty acids content

The free fatty acid (FFA) content represents the amount of potassium hydroxide (KOH) needed to neutralize the FFA in 1 g of oil. The hydrolysis of triacylglycerol and the decomposition of hydroperoxide at elevated temperatures, in the presence of air and moisture, lead to the formation of free fatty acids [4,30]. The percentage of free fatty acids in fried oil was determined according to the official method of the American Oil Chemists’ Society (AOCS Ca 5a-40, 2017) with minimal modifications based on the methods of Moufakkir et al. [31]. In this procedure, 10 g of fried oil was mixed with 2 mL of phenolphthalein solution and a few drops of 0.1 N sodium hydroxide (NaOH). The mixture was thoroughly mixed with 50 mL of ethanol until a pale pink solution was achieved. The solution was then titrated with 0.25 N NaOH, and the volume of NaOH used during titration was recorded as V_S_. For control measurements, the titration procedure was repeated using a blank solution without the sample, with the volume of NaOH used represented as V_B_. The percentage of FFA was calculated using the formula provided in equation (1):

1 $$\%\;\text{FFA}=\left(\frac{\left[\left(V_{B}-V_{S}\right)\text{mL}\;\text{of}\;\text{NaOH}\times N\times 28.14\right]}{m}\right)x100$$

where m is the mass of the test portion (g), N is the normality of NaOH, V_S_ is the volume of NaOH used in titration, and V_B_ is the volume of NaOH consumed.

#### 2.5.2 Iodine value

The iodine value (IV) is a critical metric used in the food industry to monitor the extent of hydrogenation in oils. This value quantifies the amount of unsaturated fatty acids that have reacted with iodine [[31], [32], [33]]. To determine the IV of fried oil samples treated with herb extracts, a modified titrimetric method described by Kruatian et al. [34] was employed with minor modifications. Approximately 3 g of oil was precisely weighed and placed in a 250 mL the Erlenmeyer flask. The oil was then dissolved in 25 mL of chloroform and 20 mL of Wijs solution. The flask was sealed and allowed to react for 30 min in a dark room. Subsequently, 10 mL of 15 % aqueous potassium iodide (KI) solution and 50 mL of water were added to the mixture. Titration was performed using 0.1 N sodium thiosulfate (Na_2_S_2_O_3_), and the volume of Na_2_S_2_O_3_ consumed was recorded as VS. For the control experiment, the titration procedure was repeated with a blank solution, and the volume of Na_2_S_2_O_3_ consumed was represented as V_B_. The iodine value was calculated according to equation (2):

2 $$\text{IV}=\frac{\left[\left(V_{B}-V_{S}\right)\times N\;\text{of}\;{Na}_{2}S_{2}O_{3}\right]\times 12.69}{\text{weight}\;\text{of}\;\text{sample}}$$

where V_B_ = mL Na_2_S_2_O_3_ used for blank determination, V_S_ = in the presence of air and moisture the titration procedure mL Na_2_S_2_O_3_ was used for sample determination: M = molarity of Na_2_S_2_O_3_, W = weight of the sample (g).

#### 2.5.3 Peroxid values

During frying, the thermal oxidation of oils leads to the formation of unstable peroxides and carbonyl compounds at various stages of lipid oxidation, including both primary and secondary stages. This oxidation occurs due to elevated temperatures, along with the presence of oxygen and moisture in the foods [35]. The amount of iodine produced is directly proportional to the peroxide levels, as the oxidation of unsaturated fats primarily generates peroxides [36].

The peroxide value (PV) was measured according to AOAC method 965.33 and expressed in milliequivalents of oxygen per kilogram of oil (mEq O_2_/kg). To determine the PV, 10 g of the oil sample was dissolved in 30 mL of acetic acid-chloroform (3:2) solution in a flask. Next, 1 mL of saturated potassium iodide solution was added, and the mixture was agitated until the oil was fully dissolved. The solution was incubated in the dark at room temperature for 5 min. Following this, 30 mL of distilled water was added. The solution was titrated with 0.1 N sodium thiosulfate (Na_2_S_2_O_3_) using a 1 % starch solution as an indicator until the color changed to colorless. A blank titration (without a sample) was also performed using the same method. The PV (mEq O_2_/kg) was calculated using the following equation (3):

3 $$\text{PV}=\left(\text{mEq}\;O_{2}/\;\text{kg}\right)=\frac{\left[\left(V\;–\;V_{o}\;\right)\times N\;\right]}{m}\times 1000$$

where: V = volume of Na_2_S_2_O_3_ used for titration of the sample (mL); m = mass of the sample (g)

N = the normality (mol/L) of Na_2_S_2_O_3_ V_0_ = volume of Na_2_S_2_O_3_ used for the blank (mL)

#### 2.5.4 Thiobarbituric acid reactive substances value

**Fig. 1:**
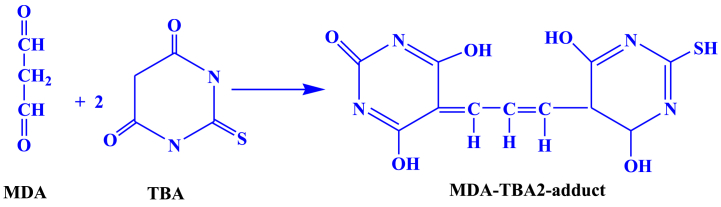
Reaction of MDA with TBA; forming MDA-TBA 2 adduct.

Malondialdehyde (MDA) is a breakdown product of an endoperoxide of unsaturated fatty acids formed when lipid substrates undergo oxidation. The quantification of Thiobarbituric acid reactive substances (TBARS) involves measuring the pink complex produced when one molecule of malondialdehyde (MDA) reacts with two molecules of 2-thiobarbituric acid (TBA) using a spectrophotometer at a wavelength of 532 nm (Fig. 1).

The TBARS value was determined following a standardized method described by Rahman et al. [37], with minor modifications. For the analysis, 5 g of samples were homogenized with 25 mL of a 20 % trichloroacetic acid solution in 135 mL/L phosphoric acid using a homogenizer for 30 s. The homogenized samples were then filtered through Whatman filter paper number 4. To 2 mL of the filtrate, 2 mL of a 0.02 M aqueous TBA solution was added in a test tube. The test tubes were incubated at 100 °C for 30 min, then cooled under running tap water. Finally, the absorbance of the supernatant solutions was measured at 532 nm using a UV–VIS spectrophotometer (UV-1200, Shimadzu, Japan). The malonaldehyde was calculated using a calibration curve established in malonaldehyde standard solutions (y = 0.1074x + 0.0143, R^2^ = 0.987), and TBARS values were expressed as milligrams of MDA per kilogram of oil (mg MDA/kg) using the following equation (4):

4 $$\text{TBARs}\;\left(\text{mg}\;\text{MDA}/\text{Kg}\;\text{of}\;\text{oil}\right)=\frac{\text{absorbance}\;x\;M.\;\text{mass}\;\text{of}\;\text{MDA}\;x\;\text{volume}\;\text{of}\;\text{extracts}\;x\;\text{DF}}{\text{sample}\;\text{mass}\;x\;\text{slop}\;\text{of}\;\text{the}\;\text{standard}\;\text{curve}}$$

#### 2.5.5 Viscosity

Viscosity is a commonly used physical parameter to evaluate the extent of frying oil deterioration in commercial and household frying experiments [38]. It also plays an important role in analyzing the stability and quality of a food system [39]. The viscosity of 100 mL of fried oil containing dietary herb extract was measured using RV spindle #3 and a 100 rpm Brookfield viscometer DV-II pro+ (Brookfield Eng. Lab., Inc., Middleboro, MA) at 40 °C, following the method described by Liu et al. [40]. The oil sample was warmed to 40 °C prior to measurement. This heating was essential to liquefy any crystals or solids that may have formed during storage.

#### 2.5.6 Color

In the food industry, color is a crucial indicator for assessing frying oil quality. This importance arises because pigment formation occurs during fatty acid oxidation and thermal decomposition, leading to the darkening of the oil [4]. Color parameters like lightness (L*), redness (a*), and yellowness (b*) are commonly used to analyze color variations between raw food materials and finished products [41]. To measure these color changes in repeatedly fried oils, the CM-200S digital color meter was employed. This high-precision device measured the color on the Lab* (CIELAB) color scale, as described by Devi [42].

### 2.6 Sensory analysis of potato chips

The sensory analysis of potato chips samples was performed using the hedonic scale as described by Serka et al. [43], with some modifications. This evaluation involved 30 panelists, consisting of 18 females and 12 males aged between 21 and 35 years. The panelists were selected from among students and academic staff in the fields of nutrition, food sciences, and technology at Hawassa University. An initial orientation session was conducted to familiarize panelists with the objectives of the study and the evaluation procedures.

Potato chip samples were cut into two pieces and arranged on service plates. Panelists were provided with water and expectoration cups to cleanse their palates between sensory assessments. Each sample tasting was randomly replicated twice by all panelists. The sensory attributes evaluated were crispiness, color, taste, and odor, and overall, accentually rated on a seven-point hedonic scale with anchor points ranging from ‘dislike extremely’ (1) to ‘like extremely’ (7). The mean scores of the sensory attributes were calculated, with a score of 5 considered the borderline of acceptability. Each data point from the sensory analysis represents the mean ranking of the 30 panelists.

### 2.7 Statistical analysis

The experimental data were analyzed using JMP Pro 13 software to evaluate differences among treatments, with significance defined at p < 0.05. The physicochemical properties of repeatedly frying oil were considered response variables. The impact of dietary herb extracts and the number of frying cycles were considered the main factors in this study. All measurements were performed in triplicate, and the results were reported as mean values.

## 3 Result and discussion

### 3.1 Physicochemical properties of repeatedly fried oil

#### 3.1.1 Change in free fatty acid content of repeatedly fried oil

**Fig. 2:**
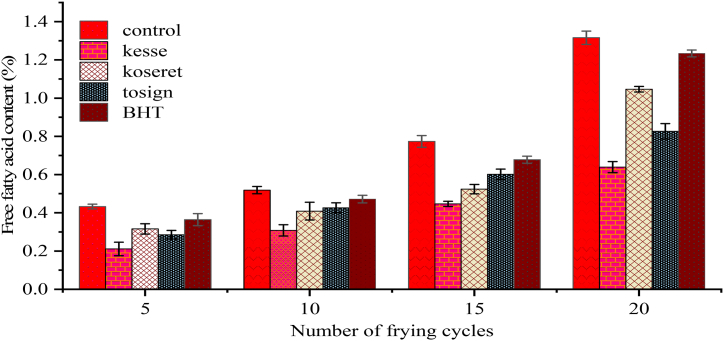
Change in free fatty acid content of repeatedly fried oils containing kesse, koseret, tosign extracts, and BHT. Values are expressed as mean ± standard deviation from three replicates.

FFA content is one of the most crucial indicators of oil quality and is used to indicate the level of hydrolytic rancidity that has occurred during frying. According to Duguma and Abebaw [44], free fatty acid (FFA) levels exceeding 1 % indicate that the frying oil has reached an exhausted state and should be withdrawn from use. The data from this study regarding the FFA values of oils subjected to repeated cycles of frying show a significant impact on the quality of the oil (Fig. 2). A continuous, significant progressive increase (p < 0.05) was observed in the FFA values of the control oil, which lacked dietary herb extracts, rising from 0.43 % in the 5th frying cycle to 1.32 % in the 20th frying cycle, equivalent to a sharp 67.42 % increase.

Among the studied herb extracts, kesse showed the most pronounced effect, maintaining the lowest FFA value across all frying cycles. Koseret and tosign extracts also result in significantly lower values of FFA with respect to the control (p < 0.05). Previous works reported that the addition of rosemary extract to sunflower oil causes a reduction in the formation of free fatty acids by about 50 % after 20 frying cycles [33]. Similarly, Turan et al. [44] reported that treatment of sunflower oil with 2000 ppm of rosemary extracts resulted in significantly lower FFA content compared to untreated oil after 20 frying cycles (p < 0.05).

Additionally, these researchers found that the levels of FFAs in a frying oil blend with different natural extracts varied between 0.08 % and 1.4 % after 12 days of use [45]. In this context, it is important to underline the positive effect of rosemary extract on sunflower oil stability during repeated frying cycles, highlighting the role of natural antioxidants in maintaining good oil quality during repeated uses [33,46].

Although the synthetic antioxidant BHT reduced FFA formation, it was not as effective as the herb extracts, particularly kesse. The rise in FFA levels during frying can be best explained by the hydrolysis of triglycerides catalyzed by available heat, water, and free fatty acids themselves. This finding aligns with existing literature that reports similar trends in FFA accumulation in oils during frying. Specifically, Mishra et al. [47] observed comparable patterns of FFA increase in repeatedly fried oils.

This can be attributed to the ability of herb extracts to inhibit hydrolytic enzymes and scavenge free radicals formed through the course of frying, as reflected in the lower FFA values observed in oils containing these herb extracts. Phenolic compounds from the herb extracts may have complexes with metal ions, leading to a reduction of their catalytic effect on hydrolysis. Additionally, the antioxidant properties of herb extracts likely hampered the thermal degradation of oil. Therefore, using dietary herb extracts in frying oils improves their quality and stability even under extended frying cycles and provides a natural alternative to synthetic antioxidants to maintain oil stability and safety.

#### 3.1.2 Change in iodine value of repeatedly fried oil

The iodine value is a widely recognized parameter in the food industry, used to assess the degree of unsaturation in fats and oils [48]. A reduction in iodine value results from complex physicochemical changes and serves as an important quality indicator during frying, reflecting the extent of oxidation [49]. Specifically, a decrease in iodine values during frying indicates an increased rate of oxidation. The study demonstrated that oils containing dietary herb extracts were more effective in preserving iodine content during frying compared to both the control and oils containing BHT (Table 1). These findings align with those of Mohd et al. [50], who reported similar positive effects with a natural antioxidant.

**Table 1:** Change in Iodine values (g I₂/100) of repeatedly fried oils containing kesse, koseret, tosign extracts, and BHT.

Treatments	Number of frying cycles			
	5	10	15	20
Control	54.30 ± 0.73^dA^	50.12 ± 0.93^dB^	46.28 ± 0.93^dC^	44.50 ± 0.75^dD^
kesse	55.63 ± 0.47^aA^	53.38 ± 0.45^aB^	48.73 ± 0.35^aC^	47.42 ± 0.40^aD^
koseret	55.25 ± 0.78^abA^	52.66 ± 0.56^abB^	48.33 ± 1.02^abC^	46.03 ± 0.68^abD^
tosign	55.09 ± 0.81^bA^	52.67 ± 0.57^bB^	48.08 ± 1.02^bC^	46.56 ± 0.48^bD^
BHT	54.8 ± 0.78^cA^	51.68 ± 0.56^cB^	46.85 ± 0.65^cC^	45.29 ± 0.52^cD^

Results are expressed as mean values ± standard deviation from three replications. Mean values within the same column that have different lowercase letters (a−c) indicate significant differences (p < 0.05). Similarly, mean values across frying cycles in the same row that have different uppercase letters (A−D) also indicate significant differences (p < 0.05).

Notably, kesse extract exhibited the highest iodine values throughout all frying cycles, suggesting its superior antioxidant properties. Koseret and tosign extracts also demonstrated significant efficacy (p < 0.05), maintaining higher iodine values than the control and showing no significant difference between each other. BHT was effective in preserving oil unsaturation, maintaining higher iodine values than the control; however, it was slightly less effective than the herb extracts, particularly kesse.

All treatments exhibited lower iodine values as the number of frying cycles increased, indicating a reduction in unsaturation due to the frying process. The control oil showed the most significant drop in iodine values, decreasing from 54.30 at the 5th frying cycle to 44.50 at the 20th frying cycle. In contrast, oils treated with kesse, koseret, tosign, and BHT showed more gradual decreases in iodine values compared to the control oil, suggesting they effectively retarded the degradation of unsaturation during frying. Specifically, the kesse-treated oil decreased from 55.63 at the 5th frying cycle to 47.42 at the 20th frying cycle. Koseret and tosign exhibited similar trends, with their iodine values decreasing to 46.03 and 46.56, respectively. BHT-treated oil decreased to 45.29 by the 20th frying cycle.

The reduction in iodine value during frying is associated with the decreased levels of unsaturated fatty acids and the effects of oxidation, as reported by Abenoza et al. [51], Chebe et al. [52], Hecke et al. [53] and Perestrelo et al. [54]. These findings are corroborated by other studies. Veronezi et al. [55] found that treating soybean oil with 3000 mg/kg of basil extract maintained oxidative stability for up to 10 h at 180 ± 5 °C. Similarly, Liu et al. [56] reported that the iodine value of oil with added antioxidants was significantly (p < 0.05) higher than that of control during the frying process, indicating that antioxidants effectively protect unsaturated fatty acids in the oil.

#### 3.1.3 Change in peroxide value of repeatedly fried oil

The peroxide value (PV) is a crucial indicator of primary oxidation compounds in oils, crucial for assessing their resistance to oxidation during frying [56]. Table 2 depicts the variations in PVs of oils treated with kesse, koseret, tosign extracts, and BHT over successive frying cycles. The data demonstrate that all treatments-kesse, koseret, tosign, and BHT−successfully preserved lower peroxide values compared to the control, implying a postponement in the commencement of oxidation and maintenance of oil quality.

**Table 2:** Change in Peroxide values (mEq O₂/kg) of repeatedly fried oils containing kesse, koseret, tosign extracts, and BHT.

Treatments	Number of frying cycles			
	5	10	15	20
Control	4.77 ± 0.34^aD^	5.58 ± 0.061^aC^	8.08 ± 0.04^aB^	9.66 ± 0.07^aA^
kesse	2.98 ± 0.14^dD^	3.46 ± 0.05^dC^	5.76 ± 0.13^cB^	6.69 ± 0.09^dA^
koseret	3.41 ± 0.04^cD^	4.37 ± 0.08b^cC^	6.52 ± 0.18^cB^	7.68 ± 0.08^cA^
tosign	2.90 ± 0.07^dD^	3.76 ± 0.07^dC^	5.87 ± 0.24^dB^	7.28 ± 0.14^dA^
BHT	4.10 ± 0.06^bD^	5.25 ± 0.08^bC^	7.52 ± 0.08^bB^	8.57 ± 0.16^bA^

Results are expressed as mean values ± standard deviation from three replications. Mean values within the same column that have different lowercase letters (a-c) indicate significant differences (p < 0.05). Similarly, mean values across frying cycles in the same row that have different uppercase letters (A-D) also indicate significant differences (p < 0.05).

Among the treatments, kesse consistently exhibited the lowest PVs throughout all frying cycles, demonstrating superior antioxidant properties that significantly delay oxidation. The oil treated with tosign extract showed comparable effectiveness, maintaining low PVs that were only slightly higher than those of kesse yet significantly lower than both the control and BHT (p < 0.05). Koser*et al*so proved to be an effective agent for sustaining low PVs compared to the control, although its values were higher than those of kesse and tosign. While BHT effectively reduced the oxidation rate compared to the control, it exhibited higher PVs than the herbal extracts.

As illustrated in Table 2, PVs increased with the number of frying cycles, indicating progressive oxidation and degradation of the oil due to the frying process. The control oil demonstrated a significant increase in PV from 4.77 at the 5th frying cycle to 9.66 at the 20th frying cycle, underscoring rapid oxidation and degradation. In contrast, oils treated with kesse, koseret, tosign, and BHT exhibited a less pronounced increase in PVs, suggesting that these treatments effectively mitigated the degradation process.

At the 5th frying cycle, kesse-treated oil exhibited a PV of 2.98, which increased to 6.69 by the 20th frying cycle. Tosign and koseret showed PVs of 7.28 and 7.68, respectively, by the 20th frying cycle. Kesse and tosign extracts demonstrated the lowest peroxide values at the 5th frying cycle, which were significantly different from those of BHT, koseret, and the control (p < 0.05). This trend persisted through subsequent frying cycles, with kesse and tosign consistently exhibiting the lowest peroxide values. Koseret displayed similar results, with no significant differences (p > 0.05) between the tosign-treated oil, but both were lower than the kesse-treated oil. The herb extracts, including BHT, were effective in reducing peroxide values; however, they were slightly higher than those of kesse and tosign extracts.

Previous studies by Guo et al. [56] and Taha et al. [57] have also demonstrated the efficacy of rosemary and thyme extracts in enhancing the oxidative stability of oils during frying. Urbančič et al. [33] reported that carnosol and carnosic acid, active ingredients in rosemary extract, effectively slowed down the deterioration of sunflower oil during deep frying and reduced acrylamide formation. Similarly, Saoudi et al. [21] evaluated the efficiency of rosemary and thyme extracts in preventing soybean oil oxidation, achieving a 70 % reduction after 24 h of heating. Zhang et al. [58] also confirmed that rosemary extracts inhibited lipid oxidation more potently than synthetic antioxidants like BHT and BHA.

#### 3.1.4 Change in TBARS of repeatedly fried oil

**Fig. 3:**
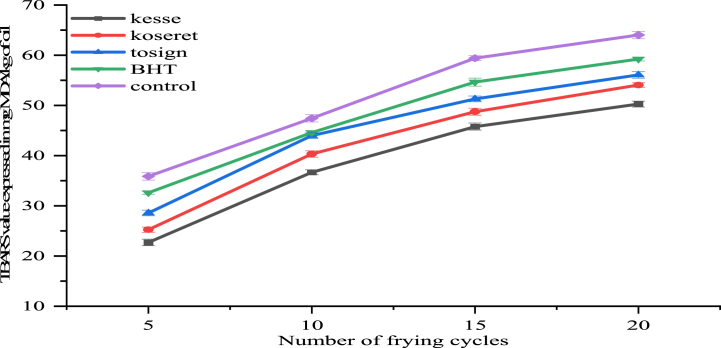
Change in TBARS value of repeatedly fried oils containing kesse, koseret, tosign extracts, and BHT. Values are expressed as mean ± standard deviation from three replicates.

The oxidation of fatty acids leads to the formation of hydroperoxide, which can further decompose into aldehydes, ketones, and acids [54]. Fig. 3 illustrated the protective effect of kesse, koseret, and koseret extracts and BHT on TBARS values (mg MDA/kg.

Oils samples containing kesse, koseret, and tosign extracts indicated a significantly lower TBARS increase with increasing number of frying number of frying cycles compared to the control oil sample, with the order of increase varying depending on the extract used. In the 5th frying cycle, oil from the control had a TBARS value of 35.88, while the oils with kesse, koseret, and tosign had significantly (p < 0.05) lower values of 22.70, 25.24, and 28.57, respectively. This trend continued up to the 20th frying cycle, where the control oil had a TBARS value of 64.02, and the kesse, koseret, and tosign extracts maintained lower values of 50.27, 54.06, and 56.08, respectively. Among the herb extracts, kesse recorded comparatively the lowest TBARS values, suggesting the better potential of this extract as far as antioxidant activity is concerned [29].

The TBARS values increased as the number of frying cycles progressed, corresponding with increased oxidative degradation and lipid peroxidation. The TBARS values in all samples significantly increased due to the progression of frying cycles. Control oil TBARS values increased from 35.88 at the 5th frying cycle to 64.02 at the 20th frying cycle. Similarly, oils containing kesse increased from 22.70 to 50.27, koseret from 25.24 to 54.07, and tosign from 28.57 to 56.08 during the same period. These results further emphasize the cumulative oxidative stress due to repeated frying, which deteriorated oil quality.

The interaction between the type of herb extract and the number of frying cycles played a critical role in influencing the TBARS values of the oils. Oils with herb extracts showed a more gradual increase in TBARS values than the control oil, suggesting that these extracts helped to mitigate the rate of lipid peroxidation over multiple frying cycles. At the 10th frying cycle, the control oil had a TBARS value of 47.42, whereas the kesse, koseret, and tosign extracts had values of 36.68, 40.31, and 43.97, respectively. The results of this study show that kesse extract is effective in suppressing the increase in TBARS, with its inhibitory effect on TBARS being more effective than that of koseret, tosign and BHT. This pattern continued through the 20th frying cycle, with the kesse extract showing the most substantial protective effect, followed by the koseret and tosign extracts. These findings suggest that dietary herb extracts and antioxidants can effectively reduce lipid oxidation, leading to lower TBARS values [53].

#### 3.1.5 Change in viscosity of repeatedly fried oil

**Fig. 4:**
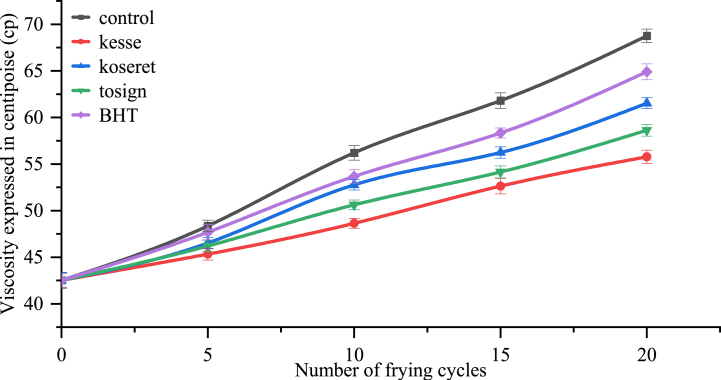
Change in Viscosity of repeatedly fried oils containing kesse, koseret, tosign extracts, and BHT. Values are expressed as mean ± standard deviation from three replicates.

The increase in viscosity during consecutive frying cycles is primarily due to the formation of various compounds via polymerization reactions, like dimers, trimers, and polymers [59]. These non-volatile polymers significantly contribute to the increasing viscosity. The study demonstrates the changes in viscosity during repeated frying cycles, particularly with the incorporation of herb extracts and BHT (Fig. 4).

In the control group, viscosity increased from 48.357 cP at the 5th frying cycle to 68.76 cP at the 20th frying cycle. In contrast, kesse extract exhibited lower viscosity levels, which increase slightly from 45.33 cP at 5th frying cycle to 55.77 cP at 20th frying cycle. Both koseret and tosign extracts had similar initial viscosities of 46.52 cP and 46.2 cP at 5th frying cycle respectively, which increase slightly to 61.53 and 58.62 by the 20th frying cycle. BHT showed a higher initial viscosity of 47.69 cP at 5th frying cycle, which increased to 64.9 cP by the 20th frying cycle, indicating a more significant change in viscosity compared to the herb extracts. The interaction of herb extracts with the frying cycles highlighted kesse's advantage, while BHT was less effective over extended frying cycles in maintaining oil quality.

The study revealed that changes in viscosity during frying cycles serve as indicators of oil degradation, which can affect the quality of fried foods [60]. Higher viscosity is associated with greater oil absorption by the product, indicating a relationship between oil quality and characteristics of the fried product. Increased degradation, shown by higher viscosity, alters the structure of the final product. Dietary herb extracts can enhance the oxidative stability of the oil [31]. As frying is a continuous process, the viscosity of the oil consistently increases, leading to the formation of denser, higher molecular weight compounds [60,61].

Certain plants with antioxidant properties are beneficial in scavenging these compounds. Among the oils treated, kesse extract demonstrated the lowest viscosity, which can be attributed to its distinct phytochemical profile. Higher viscosity is associated with higher oil content, suggesting a relationship between oil quality and the fried product. Dietary herb extracts can improve oil oxidative stability, leading to lower viscosity values. This suggests that incorporating dietary herb extracts in oil treatment can maintain lower viscosity, which can enhance oil quality during frying.

#### 3.1.6 Change in color of repeatedly fried oil

The color of oil is a critical indicator of degradation and the acceptability of food products, as highlighted by Chandran et al. [62]. Changes in oil color during frying result from chemical reactions, including hydrolysis, oxidation, and polymerization, as detailed by Nayak et al. [4]. The Hunter color scale parameters, including lightness (L*), redness (a*), and yellowness (b*), were utilized to assess color evolution. Table 3 illustrates the color profiles of oils with various herb extracts and BHT over multiple frying cycles.

**Table 3:** Color profiles (L*, b* and a*) of oil containing kesse, koseret, tosign extracts and BHT over multiple frying cycles.

Color parameter	Oil containing	Number of Frying cycles			
		5	10	15	20
L*	control	46.1 ± 1.13^cA^	35.6 ± 2.52^cB^	31.4 ± 0.53^cC^	28.68 ± 2.11^cD^
	Kesse	51.9 ± 1.38^aA^	41.3 ± 0.47^aB^	37.4 ± 1.20^aC^	36.34 ± 0.42^aD^
	koseret	49.9 ± 1.32^bA^	39.1 ± .40^bB^	35.1 ± 1.74^bC^	31.64 ± 3.16^bD^
	tosign	50.9 ± 2.81^aA^	40.2 ± 3.02^aB^	36.6 ± .29^aC^	35.17 ± 2.65^aD^
	BHT	48.7 ± 2.56^bA^	37.9 ± 1.79^bB^	33.3 ± 1.81^bC^	29.66 ± 1.48^bD^
a*	control	5.87 ± 0.03^aC^	5.94 ± 0.26^aB^	6.01 ± 0.0.01^aB^	6.57 ± 0.0.02^aA^
	Kesse	4.29 ± 0.06^dC^	4.36 ± 0.6^dB^	4.98 ± 0.01^dB^	5.56 ± 0.01^dA^
	koseret	4.71 ± 0.09^bcC^	5.40 ± 0.2b^bcB^	5.55 ± 0.10^bcB^	5.72 ± 0.10^bcA^
	tosign	4.54 ± 0.05^cdC^	5.01 ± 0.09^cdB^	5.31 ± 0.05^cdB^	5.62 ± 0.07^cdA^
	BHT	4.92 ± 0.06^bC^	5.49 ± 0.11a^bB^	5.70 ± 0.10^bB^	6.22 ± 0.09^bA^
b*	control	13.3 ± 0.82^aD^	17.3 ± 0.43^aC^	21.1 ± 1.34^aB^	29.9 ± 1.75^aA^
	Kesse	9.67 ± 0.83^abD^	13.6 ± 0.53^abC^	18.0 ± 1.22^abB^	21.8 ± 1.75^abA^
	koseret	11.1 ± 1.28^bcD^	15.2 ± 1.13^bcC^	18.5 ± 2.03^bcB^	23.9 ± 1.55^bcA^
	tosign	9.71 ± 1.46^cD^	14.3 ± 1.21^cC^	17.6 ± 1.83^cB^	22.2 ± 0.76^cA^
	BHT	11.6 ± 1.20^cD^	15.2 ± 1.24^cC^	20.8 ± 0.73^cB^	28.3 ± 0.98^cA^

Results are expressed as mean values ± standard deviation from three replications. Mean values within the same column that have different lowercase letters (a-c) indicate significant differences (p < 0.05). Similarly, mean values across frying cycles in the same row that have different uppercase letters (A-D) also indicate significant differences (p < 0.05).

The study found that oil undergoes darkening with repeated frying, as also observed by Urbani et al. [33] in their research. From the 5th to 20th frying cycles, the L* value for the control oil significantly decreased (p < 0.05) from 61.40 to 28.67. In contrast, oils treated with kesse and tosign maintained higher L* values, indicating better stability in lightness. By the 20th frying cycle, the order of the L* value was as follows: BHT-containing oil > koseret extract-containing oil > tosign extract-containing oil > kesse extract-containing oil. Notably, kesse preserved an L* value of 36.34, while the control oil dropped to 28.68.

The findings align with those reported by Oyedeji et al. [41], Ma et al. [63], Srivastava and Semwal [64], and Dodoo et al. [65], who observed a decrease in the L* values of refined coconut oil, sunflower oil, and soybean oil following repeated frying of yam chips. The darkening effect is attributed to high-temperature oil breakdown, resulting in the formation of smaller molecules that recombine into non-volatile compounds, contributing to the accumulation of free fatty acids, polymers, and oxidized triglycerides [41,63].

Additionally, the darkening can result from caramelized scorched products, which further reduce L* values of oil [7]. Mohd et al. [50] documented that the discoloration in oil samples was due to the oxidative degradation of phenolic antioxidants present during heating. The present study showed that while control oil and BHT treated oil experienced significant reductions in L* values, oils with herb extracts maintained higher L* values, demonstrating their effectiveness in preserving oil lightness over extended frying durations.

In terms of a* values, all extracts significantly reduced these values compared to the control. The control oil's a* values increased from 5.87 to 6.57, while kesse maintained lower values, increasing from 4.29 to 5.56. This suggests that herb extracts, particularly kesse, help minimize the increase in red coloration during frying.

For b* values, the control oil showed a significant increase from 13.3 to 29.9, indicating the least stability. This increase in yellowness aligns with findings in the literature, which attribute such changes to the polymerization of unsaturated carbonyl compounds and the solubilization of non-polar food compounds in the oil [64]. In contrast, oils with herb extracts maintained lower b* values, with kesse increasing from 9.67 to 21.8 and tosign from 9.71 to 22.2. A similar observation was reported in cassava chips by Oyedeji et al. [41]. The addition of BHT did not significantly change the b* value of the control oil, possibly due to the inhibition of the Maillard reaction by the extracts [66].

The frying process modifies color parameters, resulting in lower L* values and higher a* and b* values across all samples. This highlights the challenges of repetitive frying on oil quality. Importantly, the relationship between herbal extracts and frying cycles reveals a synergistic effect that enhances oil stability. Kesse and tosign-treated oils exhibited less drastic declines in L* values and smaller increases in a* and b* values, demonstrating their effectiveness in preserving color stability during frying.

### 3.2 Sensory evaluation of repeatedly fried potato chips

Studies have shown that the quality and level of acceptance by consumers for a product can be ascertained and controlled by utilizing either descriptive analysis or consumer testing to evaluate the potential impact on the overall quality or particular characteristics of the food products [65]. Potato chips are a globally beloved snack, with consumer preferences significantly shaped by sensory attributes like taste, aroma, color, and texture. The research investigated the impact of different herbal extracts and BHT on the sensory attributes of potato chips during several frying cycles. As shown in (Table 4), taste scores deceased, the control group showed a decrease in taste scores, evidenced by the scores decreasing from 5.33 points to 4.77 points by the 20th frying cycle, while kesse extract maintained the best results. Koseret and tosign had identical trends with the initial scores, dropping to around 4.80 to 4.83, showing moderate retention of sensory qualities.

**Table 4:** Sensory attributes of potato chips containing kesse, koseret, tosign extracts and BHT over multiple frying cycles.

Sensory characteristics	Treatments	Number of frying cycles			
		5	10	15	20
Taste	Control	5.33 ± 1.41^bA^	5.10 ± 1.09^bB^	4.83 ± 1.25^bC^	4.77 ± 1.12^bD^
	Kesse	5.67 ± 0.80^aA^	5.33 ± 1.35^aAB^	5.10 ± 1.07^aC^	4.93 ± 1.10^aD^
	Koseret	5.40 ± 1.31^abA^	5.13 ± 1.12^abB^	4.97 ± 1.31^abC^	4.80 ± 1.19^abD^
	tosign	5.43 ± 1.34^abA^	5.22 ± 1.08^abB^	4.97 ± 1.30^abC^	4.83 ± 1.50^abD^
	BHT	5.37 ± 1.23^bA^	5.00 ± 1.09^bB^	4.93 ± 1.42^bC^	4.77 ± 0.97^bD^
Odor	Control	5.38 ± 1.21^bA^	5.22 ± 1.00^bB^	5.00 ± 1.17^bC^	4.77 ± 0.98^bD^
	Kesse	5.67 ± 1.46^aA^	5.33 ± 1.04^aB^	5.13 ± 0.97^aC^	4.97 ± 0.99^aD^
	Koseret	5.40 ± 1.27^bA^	5.27 ± 1.07^bB^	5.07 ± 1.28^Bc^	4.83 ± 1.27^bD^
	tosign	5.43 ± 1.31^abA^	5.28 ± 1.09^abB^	5.07 ± 1.34^abC^	4.90 ± 1.35^abD^
	BHT	5.40 ± 1.21^bA^	5.25 ± 0.94^bB^	5.03 ± 1.15^bC^	4.80 ± 1.03^bD^
Color	Control	5.70 ± 0.99^dA^	5.35 ± 0.79^dA^	5.13 ± 1.04^dC^	4.67 ± 1.15^dD^
	Kesse	6.10 ± 1.49^aA^	5.67 ± 1.33^aB^	5.43 ± 1.48^aC^	5.03 ± 1.03^aD^
	Koseret	5.77 ± 1.20^bcA^	5.60 ± 1.02^bcB^	5.33 ± 1.19^bcC^	4.87 ± 1.24^bcD^
	Tosign	5.93 ± 1.45^abA^	5.62 ± 1.06^abB^	5.40 ± 1.07^abC^	4.98 ± 1.40^abD^
	BHT	5.74 ± 1.00^cdA^	5.47 ± 0.57^cdB^	5.20 ± 0.91^cdC^	4.80 ± 1.04^cdD^
Crispiness	Control	6.03 ± 0.93^bA^	5.90 ± 0.83^bA^	5.53 ± 1.21^bB^	5.20 ± .08^bC^
	Kesse	6.13 ± 1.29^aA^	6.00 ± 1.13^aA^	5.90 ± 1.10^aB^	5.60 ± 1.03^aC^
	Koseret	6.10 ± 0.97^abA^	5.97 ± 0.99^abA^	5.77 ± 1.19^abB^	5.30 ± 1.31^abC^
	Tosign	6.12 ± 1.22^abA^	5.97 ± 0.98^abA^	5.83 ± 1.16^abB^	5.47 ± 1.20^abC^
	BHT	6.03 ± 0.8^bA^	5.87 ± 0.74^bA^	5.63 ± 1.01^bB^	5.17 ± 0.76^bC^
Overall acceptability	Control	5.36 ± 1.01^bA^	5.21 ± 0.95^bB^	5.02 ± 1.14^bC^	4.93 ± 1.01^bD^
	Kesse	5.83 ± 1.42^aA^	5.57 ± 1.02^aB^	5.40 ± 0.93^aC^	5.29 ± 0.9^aD^
	Koseret	5.67 ± 0.99^abA^	5.50 ± 0.94^abB^	5.27 ± 1.10^abC^	5.13 ± 1.08^abD^
	Tosign	5.73 ± 1.21^abA^	5.52 ± 0.89^abB^	5.37 ± 1.04^abC^	5.15 ± 1.35^abD^
	BHT	5.65 ± 1.01^bA^	5.43 ± 0.86^bB^	5.27 ± 1.09^bC^	5.01 ± 0.83^bD^

Results are expressed as mean values ± standard deviation from three replications. Mean values within the same column that have different lowercase letters (a-c) indicate significant differences (p < 0.05). Similarly, mean values across frying cycles in the same row that have different uppercase letters (A-D) also indicate significant differences (p < 0.05).

For color, the control group's scores dropped from 5.70 to 4.67, while kesse consistently maintained the highest scores. Koseret and tosign showed similar trends, with scores ranging from 5.77 to 4.87 and 5.93 to 4.98, respectively. BHT scores indicated moderate enhancement in color compared to the control. Overall acceptability scores for the control group declined from 5.36 to 4.93, with kesse showing the highest scores. BHT's scores indicated some enhancement but were lower than those of kesse, tosign, and koseret. Kesse consistently provided the highest enhancement in sensory attributes, followed by tosign and koseret. BHT showed less effectiveness, while the control group showed the lowest scores. Tosign and koseret showed moderate resistance, whereas BHT was less effective. The control group showed the most significant decline, indicating that sensory attributes degraded more rapidly without additives. Overall, kesse extract was the most effective in maintaining and enhancing the sensory attributes of potato chips.

In agreement with the current study, previous studies have shown that potatoes fried in oil enriched with natural antioxidants maintained stable crispness and taste even after many frying cycles [67]. However, after the 15th frying cycle, potatoes prepared in control oil showed a significant (p < 0.05) decrease in crispness and taste. Furthermore, incorporating dietary herb extracts like *Curcuma* longa leaf extract into frying oil improved the fried potatoes’ sensory quality significantly (p < 0.05), surpassing the results of BHT and control treatments and maintaining acceptability over an extended frying period [19]. Similarly, Saoudi et al. [21] found that incorporating rosemary and thyme extracts into soybean oil significantly improved the overall acceptability of potato crisps up to the 15th frying cycle. These results underscore the effectiveness of herb extracts in enhancing the sensory qualities and overall quality of fried potatoes during prolonged frying sessions. In particular, kesse extract showed promise in improving the quality of potato chips and increasing consumer preference over extended frying periods.

### 3.3 Conclusions

This research investigated the effects of kesse, koseret, and tosign extracts, along with BHT, on the performance of frying oil and the organoleptic properties of potato chips. The results demonstrated that oils treated with herb extracts exhibited significantly better stability and quality compared to both untreated oils and BHT-treated oils. Notably, kesse and tosign extracts resulted in lower free fatty acid values, suggesting improved maintenance of oil quality during frying. Additionally, the higher iodine values obtained for herb-treated oils indicate better retention of unsaturation, which is crucial for oil stability. Among the extracts, kesse exhibited the lowest peroxide value, indicating superior protection against oxidation. The extracts also helped preserve the viscosity and lighter color of the oil, both of which are important for the visual appeal of fried potato products. The sensory evaluation test results indicated that potato chips fried in oils with herbal extracts were preferred in terms of taste, aroma, color, and overall acceptance. The finding highlights the potential of kesse, koseret, and tosign extracts as natural alternatives to synthetic antioxidants for enhancing the quality and stability of frying oils. Further research is needed to examine the effect of frying temperatures on the stability of the bioactive compounds in these herbs. Additionally, optimizing extraction methods and exploring their use in cooking processes are necessary to establish these extracts as effective natural preservatives for edible oils.

## Ethics Statement

This study was conducted in accordance with the ethical guidelines approved by the School of Food Science and Technology at Hawassa University, Ethiopia. Written informed consent was obtained from all participants involved in the sensory evaluation. The study protocol was reviewed and approved by Hawassa University, ensuring compliance with ethical standards.

## Data availability Statement

The data that support the findings are available on request from the corresponding author.

## CRediT authorship contribution statement

**Daniel Assefa:** Writing – original draft, Visualization, Software, Resources, Methodology, Investigation, Formal analysis, Data curation, Conceptualization. **Engida Dessalegn:** Writing – review & editing, Visualization, Validation, Supervision, Software, Methodology, Conceptualization. **Kebede Abegaz:** Writing – review & editing, Visualization, Validation, Software, Methodology, Formal analysis, Data curation, Conceptualization.

## Declaration of competing interest

The authors declare that they have no known competing financial interests or personal relationships that could have appeared to influence the work reported in this paper.
